# Methods for neuronal guiding and synapse formation

**Published:** 2012-06-18

**Authors:** DD Banciu, A Marin, BM Radu

**Affiliations:** * “Carol Davila” University of Medicine and Pharmacy, Bucharest, Romania; ** University of Bucharest, Faculty of Biology, Department of Anatomy, Animal Physiology and Biophysics, Bucharest, Romania

**Keywords:** neuronal growth, synapse formation, laser guiding, optical tweezers

## Abstract

**Hypothesis:** Light at certain wavelengths, can cross the cell, tissue sections and organ tissues for in-vitro studies, but can also be used for in-vivo studies. The ability to guide the growth of neural extensions was proven by laser. Synapses produced with laser have the instability as main disadvantage, in the absence of high densities of neurons. The main mechanism of synapse formation is the synaptic plasticity. In this case, dendritic learning spines increase in size and are transformed in dendritic memory spines.

**Aim:** The goal of this study was to evaluate if the creation of synapses using laser, without cell disruptions, is feasible.

**Methods and Results:** We have stimulated neuronal guiding growth using a laser system called multipoint optical tweezers. Approaches were made between dendrites and neuronal bodies. Normal mechanism of synapses formation was stimulated by using electrical stimuli, applied by using a patch-clamp set-up. This approach revealed the transmission of electrical stimuli on both sides of the synapse, but also its unidirectional transmission, which is correlated with cell integrity.

**Discussion:** The feasibility of achieving laser synapses was proven, and this method can be useful for the development of neural circuits, and for the modulation of the existing ones. We suggest that light is the best tool to guide neural growth and synapse formation. This method is cost efficient and very easy to perform. Our model is very good for understanding molecular mechanisms in pathological neurons that are relocated in key positions from pathological tissues or from transfected cell lines.

## Introduction

Neuroscience is a fast growing research field interdependent with borderline research areas. Biophysics and biotechnology offer tools for studying neurons from single cell to more complex intercellular interactions. It represents a gap between researchers from different fields that requires predictability for scientists who can design a new method to investigate neural networks. If research of single cell is covered by equipment, interactions between cells are limited to self-assembling interactions or slice from brain. 

For the better understanding of neurons in single cell and interactions in neural network, we need to be able to design guided neural networks that can answer to specific questions. It is possible to create neural networks on predesigned patterns, but we are limited by insufficient research tools that can be provided by applied research fields. 

Neural networks models are very important for pharmacologic industry. Drug design process needs an important step of testing on similar models in order to observe different actions of proposed active compounds [**1**]. In order to increase the speed of this process, it is necessary to run a huge number of tests that require an equivalent number of measurement set-ups [**2**]. Optical investigation methods have accuracy and are relatively non-expensive [**3**]. Interaction between light and living cells can be mediated by small mechanisms that are inspired from Mother Nature.

Neural networks can give an important alternative for computing in nuclear radiation area. Living cells have a capacity to auto repair [**4**] that cannot be challenged by any human designed computer. In order to increase resistance to nuclear radiation we can transfect factors from radio resistant tumors, or use neuron lines from scorpions. There are many well-known examples of radio resistance. Nuclear research can benefit from tools designed this way. 

Researches in guided neural growth show that is possible to use light to manipulate the way of neural development. This is important for the construct predesigned neural pathways from individual cells to almost connected cells [5]. Neurons can make synapses randomly, but only frequently used synapses have a long lifetime [**5**]. Small synaptic spins, which can be formed without environmental stimulation, represent short lifetime interneuron connection [**6**]. If synapse is efficient in transmitting signals, these spins can be transformed in large spins with long lifetime [**7**]. 

From these considerations, neural networks constructed this way, need an auto-oscillatory design for self-organizing after guided neural growth [**8**]. These oscillators can be inhibited or stimulated by environmental factors, locally or externally. It is possible to create logical circuits that can be assembled in more complex biological computer. 

With this model, we propose not only a way to investigate neurons, but also related research fields.

## Aim

The punctual issue of this paper focuses on the construction of synapse with lasers. This approach was inspired as an element of Ehrlicher experiments published in 2002 [**8**], which revealed the possibility to guide the growth of neuronal extensions with lasers.

## Methods

Dorsal root ganglia neurons culture

Adult male Wistar rats (150-200 g) were anesthetized by inhalation of 100% CO2 (2 min exposure) followed by decapitation. The procedures were conducted in accordance with the Guidelines of University of Bucharest regarding the care and use of animals for experimentation. Dorsal root ganglia were removed as previously described [**9**]. They were incubated for 1h at 37ºC in IncMix solution containing (in mM): NaCl 155; KH2PO4 1.5; HEPES 5.6; NaHEPES 4.8; glucose 5, 50 μg/ml gentamicin, 1 mg/ml collagenase (type XI, Sigma) and 1 mg/ml dispase (Gibco). Neurons were dissociated by trituration and plated for 1h on Petri dishes (Corning) treated with poly-D-Lysine (0.1 mg/ml, 30 min) and were cultured at 37ºC in DMEM and Hams’s F10 medium with 10% horse serum and 50 μg/ ml gentamicin, 5% CO2.

Solutions

The extracellular solution contained (in mM): 140 NaCl, 4 KCl, 2 CaCl2, 1 MgCl2, 10 HEPES (pH 7.4 with NaOH at 25ºC) to which glucose (5 mM) was added on the day of the experiment. 

The intracellular solution contained (in mM): 120 KCl, 10 NaCl, 10 BAPTA / 24.2 KOH, 10 HEPES, 1 CaCl2, 3.45 MgCl2, (pH 7.2 at 25ºC with KOH) and filtered at 0.2 μm immediately before use. All the reagents were purchased from Sigma.

Electrophysiological recordings

Electrophysiological characterization of neurons in primary culture was performed by whole-cell patch-clamp technique. Borosilicate micropipettes were prepared at a resistance of 3-7 MΩ with a P-30 vertical pipette puller (Sutter). Whole-cell recordings were made with Axopatch 200B (Axon Instruments). Stimuli were controlled and digitized with pClamp 10.0 and Digidata 1440 interface (Molecular Devices). Series resistance (Rs) was compensated (40-60%) with the patch-clamp amplifier. Double patch-clamp recordings were used to evaluate synapses. Synaptic connections were detected in current-clamp mode by stimulating the presynaptic neuron depolarization by injecting a rectangular current of 100-300 pA with duration of 1 s and recording in the postsynaptic neuron the depolarizing postsynaptic potential. The protocol was repeated in reverse order, stimulating the postsynaptic neuron and recording presynaptic neuron.

Neural guiding and synapse formation

Laser stimulation of neuronal extensions is a common method of guiding neuronal growth. This is dependent on the wavelength, to a degree that cannot be presently evaluated. Stimulation was performed at a wavelength of 1064 nm. Laser stimulation was made by using the optical tweezers set, mounted on an AxioObserver D1 (Zeiss). Neuronal extensions were guided to the proximity of neuronal bodies, in order to optimize spatial formation of synapses.

## Results

**Fig. 1:**
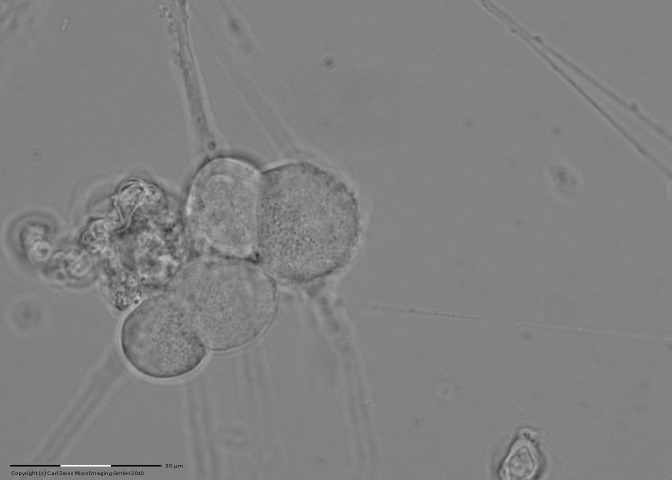
Neuronal extension with an asymmetry, selected to stimulate the laser (on the right side of image)

It starts from a cell that already has an extension of neuronal, possibly an extension of neuronal asymmetry and laser stimulation, in order to guide neural growth (**Fig. 1**).

**Fig. 2:**
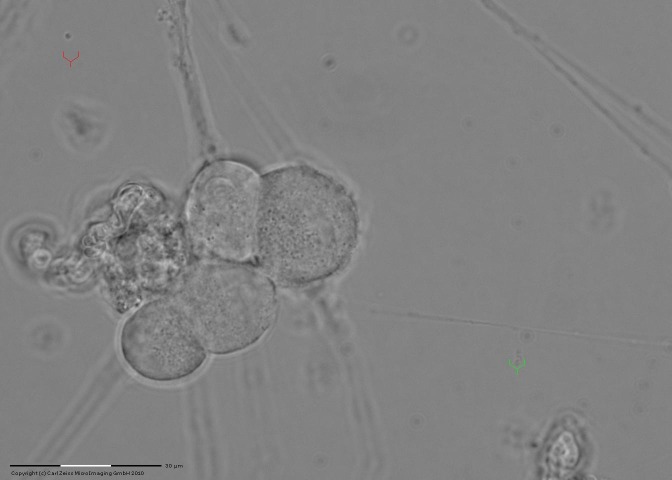
Increased neuronal extensions after laser stimulation (direct method of evidence) and present position Y-shaped optical trap. Since laser light is not visible, the Zeiss system uses a Y-shaped optical trap (marked in green). Compared with **Fig. 1**, which highlights the optical guided neuronal growth, from neuronal asymmetry up to neuronal extension.

After laser pulse stimulation, we noticed an increase in neuronal extension, in the direction of optical tweezers (**Fig. 2**), within 2 minutes from the previous image. 

**Fig. 3:**
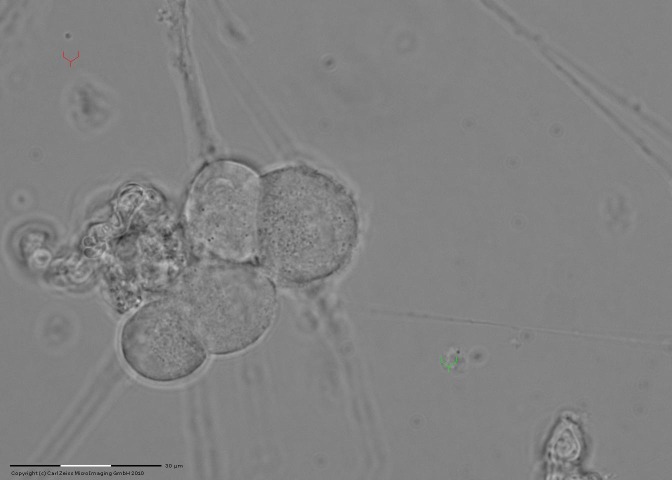
Pointing out the guided unadherent extension to substrate (indirect method of evidence)

To reflect the substrate adhesion of the neural guided growth, guidance is accomplished correlated with changing direction of extension growth by stimulating the X-axis (to the left), for a period of about 1 minute (**Fig. 3**).

Highlighting the neuronal extension is made directly by viewing its path, but also indirectly by simultaneous movement of the optical trap particles adhered to the extension. Often, indirect pointing is easier, and therefore it was necessary to test the lateral movement in the substrate adhesion of laser guided neuronal extension.

Guiding neural growth, which was able to lead to the formation of synapses, is directly related to local concentration of neurons. This may be due to intercellular NGF secreted by neurons. 

The limitations of the method are multiple, one of the most important one being the rate of reproducibility. In our system, the optical guiding has a reproducibility of 100% neuronal growth, and, the rate of synapse formation was of 12 out of 40 experiments. 

**Fig. 4:**
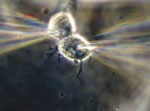
Double patch-clamp experiment. The optical focus was chosen to highlight the cells and the patch-clamp pipettes prepared from glass capillaries.

An important characteristic of laser radiation is the ability to perform photoporation. This provides a solution of continuity between the extracellular and intracellular environment. Synapse formation by neuronal growth guidance may lead to mechanical tension in the membranes of neuronal extensions, with an increase in their fragility. We assessed its unidirectional transmission to demonstrate if there was a synapse or a gap type connection mediated by photoporation between the two cells (**Fig. 4**).

**Fig. 5:**
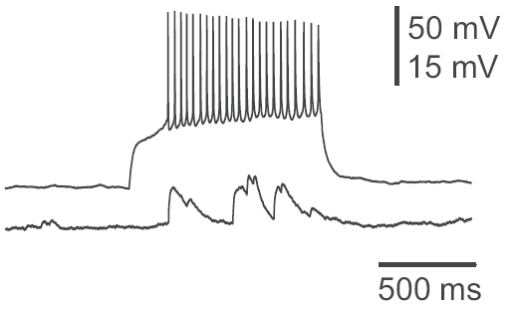
The recording of a local potential in the postsynaptic neuron after stimulation of presynaptic neuron

**Fig. 6:**
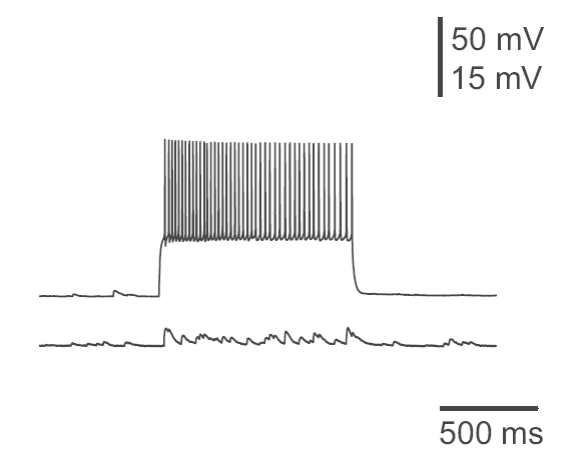
The recording of a local potential in the presynaptic neuron after stimulation of postsynaptic neuron

Initially, the neuron with optical guided neuronal growth was electrically stimulated, and neuronal activity was recorded on target neuron (**Fig. 5**), and then vice versa (**Fig. 6**). The existence of chemical synapses mediated by optical guided synapse formation was confirmed by unidirectional transmission.

## Discussion

Laser light is a great tool, but it is not used at its full capacity in medicine. It was proved that the creation of synapses using laser, without cell disruptions, is feasible. Synapse function was evaluated by electrophysiological methods, which allowed an assessment of cellular integrity by using the unidirectional transmission of the electrical stimulus (**seeFig. 4, Fig. 5**).

Direct clinical application of this study is much easier to achieve under current technology by using optical guidance. In addition, it is important to note that our study focused on neuronal growth guided in primary cultures obtained from adult rats, and not from embryos. This is important because synaptogenesis is greatly increased during the embryonic stage compared to adult stage, but, the most common in clinical situations involving the recovery and rehabilitation in adult nerves. Our study can lead to advanced development of biotechnologies for clinical applications.

**Acknowledgements**

The authors gratefully acknowledge Prof. Dr. Doina Dumitrescu Ionescu, Prof. Dr. Eugenia Kovacs, Assoc. Prof. Dr. Tudor Savopol, Dr. Mihai Radu, Assoc. Prof. Dr. Bogdan Amuzescu for valued advice. 

**Sources of Funding** This work is supported by grant PN II 61-011/2007. This work was supported by the strategic grant POSDRU/89/1.5/S/58852, Project “Postdoctoral programme for training scientific researchers” co-financed by the European Social Found within the Sectorial Operational Program Human Resources Development 2007-2013.

**Disclosures**

Banciu Daniel Dumitru, Marin Adela, Radu Beatrice Mihaela, “Carol Davila” University of Medicine and Pharmacy, Bucharest, have intellectual property rights that could be perceived as real or apparent conflict(s) of interest. These property rights began to be protected by the patent application, published in the Official Industrial Property Bulletin (BOPI) of State Office for Inventions and Trademarks (OSIM).
